# Evaluation of the Nutritional Quality of Chinese Kale (*Brassica alboglabra* Bailey) Using UHPLC-Quadrupole-Orbitrap MS/MS-Based Metabolomics

**DOI:** 10.3390/molecules22081262

**Published:** 2017-07-27

**Authors:** Ya-Qin Wang, Li-Ping Hu, Guang-Min Liu, De-Shuang Zhang, Hong-Ju He

**Affiliations:** Beijing Vegetable Research Center, Beijing Academy of Agriculture and Forestry Sciences, Beijing 10097, China; wangyaqin@nercv.org (Y.-Q.W.); huliping@nercv.org (L.-P.H.); liuguangmin@nercv.org (G.-M.L.); zhangdeshuang@nercv.org (D.-S.Z.)

**Keywords:** Chinese kale, nutritional quality, metabolomics, Quadrupole-Orbitrap MS/MS

## Abstract

Chinese kale (*Brassica alboglabra* Bailey) is a widely consumed vegetable which is rich in antioxidants and anticarcinogenic compounds. Herein, we used an untargeted ultra-high-performance liquid chromatography (UHPLC)-Quadrupole-Orbitrap MS/MS-based metabolomics strategy to study the nutrient profiles of Chinese kale. Seven Chinese kale cultivars and three different edible parts were evaluated, and amino acids, sugars, organic acids, glucosinolates and phenolic compounds were analysed simultaneously. We found that two cultivars, a purple-stem cultivar W1 and a yellow-flower cultivar Y1, had more health-promoting compounds than others. The multivariate statistical analysis results showed that gluconapin was the most important contributor for discriminating both cultivars and edible parts. The purple-stem cultivar W1 had higher levels of some phenolic acids and flavonoids than the green stem cultivars. Compared to stems and leaves, the inflorescences contained more amino acids, glucosinolates and most of the phenolic acids. Meanwhile, the stems had the least amounts of phenolic compounds among the organs tested. Metabolomics is a powerful approach for the comprehensive understanding of vegetable nutritional quality. The results provide the basis for future metabolomics-guided breeding and nutritional quality improvement.

## 1. Introduction

*Brassicaceae* vegetables have long been recognised for their nutritional attributes. Numerous epidemiological studies indicate that the consumption of *Brassicaceae* vegetables is linked to a reduced incidence of cancer and cardiovascular diseases [1]. Chinese kale (*Brassica alboglabra* Bailey) is an original Chinese vegetable belonging to the *Brassicaceae* family. It is widespread in China and Southeast Asia, with a large growing area and a marketable supply in these regions [2]. Generally, Chinese kale is consumed for its bolting stems as common edible parts, and the tender rosette leaves are also widely consumed as a leafy vegetable. Chinese kale exhibits a high nutritional value since it is a rich source of antioxidants and anticarcinogenic compounds, including vitamin C, glucosinolates and phenolic compounds [3]. Vitamin C (l-ascorbic acid) is an important primary metabolite of plants, functioning as a powerful antioxidant, an enzyme cofactor, and a cell-signalling modulator in a wide array of crucial physiological processes [4]. Glucosinolates (GSLs) are a group of sulphur- and nitrogen-containing secondary metabolites that are mainly found in the *Brassicaceae* family. Epidemiological studies have shown that the hydrolysed products of GSLs could protect humans against different types of cancer [5,6]. Sulforaphane, the active hydrolysis compound of glucoraphanin, is a strong naturally occurring inducer of phase II enzymes that detoxify carcinogens [7]. Phenolics have received considerable attention due to their potential human health-promoting effects, including antioxidant, anti-inflammatory, antimicrobial, antiallergic and anticarcinogenic capacities [8].

Because health-promoting characteristics of food are increasingly in demand and are included in purchasing decisions by consumers, the beneficial properties of Chinese kale suggest the importance of measuring the composition of its bioactive compounds. As we know, the composition of bioactive compounds in vegetables can be strongly influenced by various biotic and environmental factors, such as genotype, tissue, developmental stage, climate condition, fertilisation, storage and processing conditions [9,10,11,12,13]. Understanding the metabolite profiles of edible plants under different biotic and abiotic conditions is crucial for designing strategies to enhance their nutritive value. The profiles of GSLs or phenolic compounds in *Brassicaceae* vegetables have been extensively explored. With regard to Chinese kale, the variations of GSLs among different varieties and edible parts were investigated [2,14]. Effects of plant hormones on the contents of GSLs in Chinese kale were also investigated [3]. However, only limited information is available for the phenolic profiles of Chinese kale so far. There is still no comprehensive study on the nutrient profile of Chinese kale, which would help in the selection of cultivars with higher contents of health-promoting compounds and in designing strategies to enhance their nutritive value.

With the development of analytical technology, the study of the influence of different factors on plant metabolites is no longer limited to a series of targeted compounds. The comprehensive understanding of the entire metabolic network and a simultaneous analysis of thousands of metabolites are necessary. Metabolomics is a valuable technology for the comprehensive profiling and comparison of metabolites in biological systems. In particular, metabolomics has proved to be a useful approach for plants, where thousands of unknown metabolites occur with high diversity and in a wide range of concentrations [15]. The untargeted strategy, as an unbiased approach, could be used for comparing profiles of metabolites between groups and helps researchers better understand the complexity of these mixtures [16]. It could also contribute to identifying the metabolites that might play the most relevant role in various processes [17]. Metabolomic approaches, especially LC-MS-based metabolomics, have been successfully applied to study the influence on *Brassicaceous* vegetables quality of biotic and abiotic factors [18], including genotypic variation and tissue differences [19,20,21], environmental factors and cultivation practice influences [16,22], and post-harvest processing method effects [23]. Metabolomics has been shown to be an important and irreplaceable tool for the improvement of vegetable nutritive value.

When performing LC-MS-based metabolomics, metabolite identification represents an issue because spectral libraries are currently limited [24]. Metabolite identification relies on the comparison of the experimental accurate mass with those in available databases. High mass accuracy is a prerequisite for effective database searching and reliable identification. The Orbitrap is a mass analyser of the Fourier transform family. It routinely provides a high and reproducible mass accuracy in combination with sufficient dynamic mass range and scan speed [25,26]. It has been used for a comprehensive profiling of polyphenols in five *Brassicas* (mizuna, red cabbage, purple kohlrabi, red mustard and purple mustard) at their microgreen growth stage [27]. Besides, the analyser has also been used in the metabolomic study of plants such as barley [28], chili pepper [29] and traditional Chinese medicine [30]. The Orbitrap is a useful and reliable tool for non-targeted plant metabolomic study and is becoming a mainstream instrument in LC-MS-based metabolomics [31].

In the present study, we aimed to use the untargeted metabolomics strategy to study the nutrient profiles of Chinese kale. Based on previous breeding work of Chinese kale in our institution and a comprehensive evaluation of vegetable shape, taste and yield, seven Chinese kale cultivars were chosen to investigate their nutritional quality. An ultra-high-performance liquid chromatography (UHPLC)-Quadrupole-Orbitrap MS/MS method was employed. The important metabolites featuring cultivars were identified. We also compared the metabolite profiles of different edible parts of Chinese kale in order to provide data for guiding human consumption. Our systematic study will add more understanding on the nutrient profiles of Chinese kale and provide a foundation for improving vegetable nutritional quality in the future.

## 2. Results and Discussion

### 2.1. Metabolic Profiles of Different Chinese Kale Cultivars

The whole edible parts from seven Chinese kale cultivars, including five white flower cultivars (W1, W2, W3, W4 and W5) and two yellow flower cultivars (Y1 and Y2), were harvested and the metabolites were extracted. The metabolite profiles of Chinese kale were investigated using the UHPLC-Quadrupole-Orbitrap MS/MS-based method. After pre-processing the raw data, a total of 756 nonredundant features were obtained from the whole edible parts of all seven Chinese kale cultivars, and 726 of them were significantly different (*p* < 0.05) among seven cultivars. To obtain an overview of the sample distribution and highlight discriminant analytes, the data that passed the analysis of variance (ANOVA) screen were further explored using orthogonal partial least squares-discriminate analysis (OPLS-DA). Variables with high relevance for explaining the differences among cultivars were identified. A total of 104 features were screened with a variable importance for the projection (VIP) score higher than 1, 47 of which were assigned. The tentatively identified compounds included amino acids, organic acids, sugars and derivatives, vitamins, GSLs, phenolic acids and flavonoids. The retention time, molecular formula, accurate mass and fragment ions were listed in Table 1. The assigned compounds with high VIP values (VIP > 3) were gluconapin, glucoraphanin, citric acid, malic acid, chlorogenic acid, 4-methoxyglucobrassicin, neoglucobrassicin, glucobrassicin, sinigrin and glutamine. These compounds were considered to be the most contributed features for distinguishing different Chinese kale cultivars.

**Figure 1:**
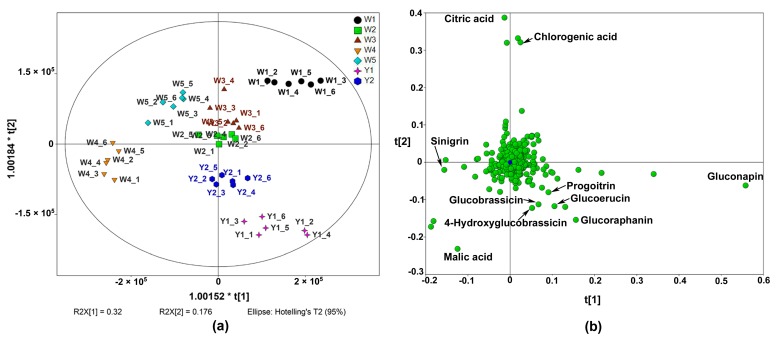
Orthogonal partial least squares-discriminate analysis (OPLS-DA) of seven Chinese kale cultivars analysed by UHPLC-Quadrupole-Orbitrap MS/MS (the number after the cultivar name stands for the six independent biological replicates of each cultivar). (**a**) Score plot; (**b**) Loading plot.

The score plot of the OPLS-DA (Figure 1a) revealed a clear separation among cultivars. Basically, the five white-flower cultivars were separated along the first component, with W1 on the positive side and W4 on the negative side. Gluconapin, glucoraphanin and glucoerucin had higher loading scores in component 1 (Figure 1b). It indicated that these GSLs were more abundant in W1 than other white-flower cultivars. Sinigrin and malic acid could be identified with higher loading scores on the negative side of the first component, i.e., with higher levels in W4. W2 and W3 were located close to the origin, and they had similar metabolite profiles. The two yellow-flower cultivars were located on the lower-right of the score plot, distinct from W1, W2, W3 and W5 along the second component. The positive side of the second component was characterised by citric acid and chlorogenic acid. Meanwhile, malic acid, glucoraphanin, glucoerucin, 4-hydroxyglucobrassicin and glucobrassicin had higher loading scores on the negative side of component 2, and these compounds were higher in Y1.

### 2.2. Nutritive Compounds in Different Chinese Kale Cultivars

#### 2.2.1. Primary Metabolites in Different Chinese Kale Cultivars

**Figure 2:**
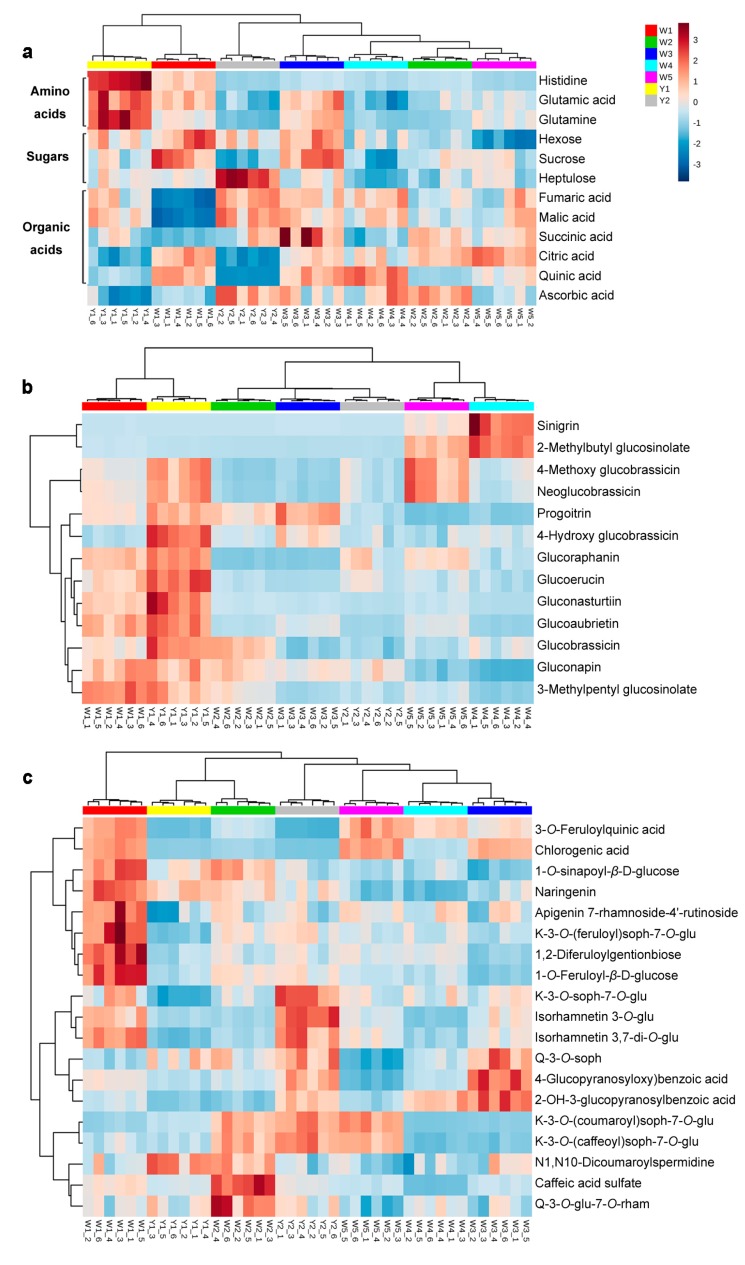
Hierarchical clustering analysis (HCA) of the primary and secondary nutritive metabolites identified in different Chinese kale cultivars (each cultivar with six independent biological replicates). (**a**) Primary metabolites; (**b**) Glucosinolates; (**c**) Phenolic compounds. glu: glucoside; rham: rhamnoside; K: kaempferol; Q: quercetin; soph: sophoroside.

The differences in identified nutritive compounds among cultivars were further analysed using hierarchical clustering analysis (HCA), and the result is shown in Figure 2.

The primary metabolites identified included three amino acids (glutamic acid, glutamine and histidine), three sugars (hexose, sucrose and heptulose), five organic acids (fumaric acid, malic acid, succinic acid, citric acid and quinic acid) and vitamin C (ascorbic acid) (Figure 2a). Y1 was distinct on the basis of high levels of amino acids. Regarding the sugars, W1 and W3 contained higher levels of hexose (glucose/fructose) and sucrose, while Y2 contained more heptulose. Y2 and W3 had higher levels of fumaric acid, malic acid and succinic acid. The yellow-flower cultivars Y1 and Y2 had lower levels of citric acid and quinic acid. In sum, W1 and Y1 had more similar primary metabolite profiles, which were distinct from those of W2, W4 and W5. In addition, Y2 and W2 had higher levels of vitamin C compared to the others.

#### 2.2.2. Glucosinolates in Different Chinese Kale Cultivars

Thirteen GSLs were tentatively identified, including seven aliphatic GSLs (gluconapin, glucoraphanin, sinigrin, progoitrin, glucoerucin, 2-methylbutylglucosinolate and 3-methylpentylglucosinolate), four indolyl GSLs (glucobrassicin, 4-hydroxy glucobrassicin, 4-methoxy glucobrassicin and neoglucobrassicin) and two phenyl GSLs (gluconasturtiin and glucoaubrietin). Nine of these GSLs were further quantified, including five aliphatic GSLs and four indolyl GSLs. The quantification result is shown in Table S1. In general, most of the GSLs were more abundant in Y1 except for sinigrin and 2-methylbutyl glucosinolate, which were higher in W4 (Figure 2b). In comparison, Y2 contained much less GSLs. The flower colour exerted little influence on the composition of GSLs in all cultivars, which was consistent with previous studies [14].

According to the result of the OPLS-DA, the most contributed compounds for cultivar discrimination were screened (Figure 1b), and most of them were GSLs, especially gluconapin, which had the highest VIP score of all the significantly different features among cultivars. It was reported that aliphatic GSLs were predominant in Chinese kale, with gluconapin being the most abundant [3,14]. In the present study, the aliphatic GSLs accounted for more than 80% of the total GSLs quantified. 4C GSLs (glucoraphanin and gluconapin) were the major GSLs in Chinese kale, as previously reported by Sun et al. [2]. W1 and Y1 had much higher levels of gluconapin compared to the other cultivars. Glucoraphanin, the precursor of gluconapin in the GSL biosynthesis pathway [32], was also abundant in these two cultivars, and they contained more than ten times higher glucoraphanin than W2 and W3 (Table S1). Of the indolyl GSLs, glucobrassicin was dominant in each cultivar, whereas other indolyl GSLs were found in low amounts.

The breakdown products of gluconapin are associated with the flavour of pungency and bitterness [14,33]. However, several hydrolysis products of GSLs such as glucoraphanin, glucoiberin and glucobrassicin are considered to reduce the risk of cancers [5,7,34]. Sulforaphane, the active hydrolysis compound of glucoraphanin, is a strong naturally occurring inducer of phase II enzymes that detoxify carcinogens. From the nutritional and sensory perspectives, the cultivars with high contents of health-promoting GSLs that do not contribute to undesirable flavour attributes could be ideal materials for future breeding programmes [35]. Understanding GSL biosynthesis provides a potential approach to alter levels of specific GSLs in plants via metabolic engineering [14,36]. The biosynthetic pathway of aliphatic GSLs has been basically elucidated. Three important genetic loci (*GSL-OX*, *GSL-ALK*, and *GSL-OH*), identified in *Brassica* species and *Arabidopsis*, contribute to the clarification of the side chain modification pathway of aliphatic GSLs [14,36]. Qian et al. [14] has focused on the function of the *AOP2* gene (at the *GSL-ALK* locus), which is involved in the conversion of glucoraphanin to gluconapin, and they found a higher content of glucoraphanin in the antisense *AOP2* transgenic Chinese kale. More investigations are needed to clarify the regulation mechanism involved. A cultivar with a rich abundance of anticancer GSLs, such as Y1, could be a good candidate for further metabolic regulation studies.

#### 2.2.3. Phenolic Acids and Flavonoids in Different Chinese Kale Cultivars

Phenolic acids and flavonoids are the most characterised groups of phenolic compounds in *Brassica* [20]. A total of nine phenolic acids and ten flavonoids were identified (Figure 2c). The cultivars with the same flower colour did not show similar phenolic profiles. The three white-flower cultivars, W3, W4 and W5, had similar phenolic compound profiles. Y2 was clustered closely to them, but it contained more flavonoids, such as kaempferol 3-*O*-sophoroside-7-*O*-glucoside, isorhamnetin 3-*O*-glucoside and isorhamnetin 3,7-di-*O*-glucoside. Another white-flower cultivar, W2, was clustered with the yellow-flower cultivar Y1 as a group, while the phenolics were higher in W2.

The phenolic compound profiles of the purple-stem cultivar W1 differed from those of the green-stem cultivars (Figure 2c). W1 contained much higher levels of five phenolic acids and four flavonoids, including 3-*O*-feruloylquinic acid, chlorogenic acid, 1-*O*-sinapoyl-*β*-d-glucose, 1-*O*-feruloyl-*β*-d-glucose, 1,2-diferuloylgentionbiose, naringenin, apigenin 7-rhamnoside-4′-rutinoside, kaempferol 3-*O*-(feruloyl)-sophoroside-7-*O*-glucoside and isorhamnetin 3,7-di-*O*-glucoside. It was reported that the total phenolic acid contents were more than six-fold higher in red cabbages than those in green cabbages [20]. In the present study, these phenolic compounds were considered to contribute to a different stem colour phenotype. The flowers were a minor portion of the whole edible part compared to stems (Figure S1). The pigments in the flowers were not sufficient to be distinct metabolite features for cultivar separation.

The data about the phenolic compound profiles of Chinese kale are limited so far. Many of the phenolic compounds, including their glycosides compounds, were previously reported in other *Brassica* vegetables [37]. Phenolic acids were abundant in most common vegetables, mainly as sulfate and glucuronate derivatives [38]. The majority of flavonoids in Chinese kale are flavonol glycosides, mainly composed of kaempferol and quercetin. Lin and Harnly [37] reported that isorhamnetin was the only other flavonol to form glycosides in Chinese kale. In the present study, apigenin 7-rhamnoside-4′-rutinoside was also tentatively identified according to its accurate mass and MS/MS fragments (Table 1).

Phenolic compounds received special attention because of their relatively high concentrations in vegetables and human health-promoting effects. The purple-stem cultivar W1 was a good source of phenolics. Moreover, W1 also contained high levels of amino acids, sugars, gluconapin, glucoraphanin and glucoerucin. From the nutritional point of view, W1 may be highly recommended. Together with Y1 which contained a rich content of GSLs and high levels of amino acids, these two cultivars possessed higher nutritive values than the others.

In summary, the metabolite profiles of seven Chinese kale cultivars were obviously different. Two cultivars, W1 and Y1, had more amino acids and GSLs than others. The purple-stem cultivar W1 was rich in phenolic acids and flavonoids. These cultivars could be good candidates for consumption and for further studying of metabolic regulation.

### 2.3. Metabolic Profiles of Different Edible Parts of Chinese Kale

The metabolite profiles of different edible parts of Chinese kale, including the leaves (L), stems (S) and inflorescences (F), were investigated. All of these parts as a whole (E) were also evaluated since this is the typical way people consume Chinese kale. Three white-flower cultivars (W1, W2 and W5) were chosen because white-flower cultivars are the majority of Chinese kale in market supply and consumption. A total of 805 frames had significantly different levels (*p* < 0.05) among the four groups. Then, the data were handled with OPLS-DA to discriminate sample groups. A total of 132 features were screened with VIP > 1, and 48 were tentatively identified. Gluconapin, citric acid, glutamine, N1,N10-dicoumaroylspermidine, quercetin 3-*O*-glucoside-7-*O*-rhamnoside, glucobrassicin, ascorbic acid, malic acid and apigenin 7-rhamnoside-4′-rutinoside were the most distinguished metabolites among the groups (VIP > 2.5). Gluconapin was also the compound with the highest VIP value, and was considered to be the most distinguished metabolite among edible parts.

**Figure 3:**
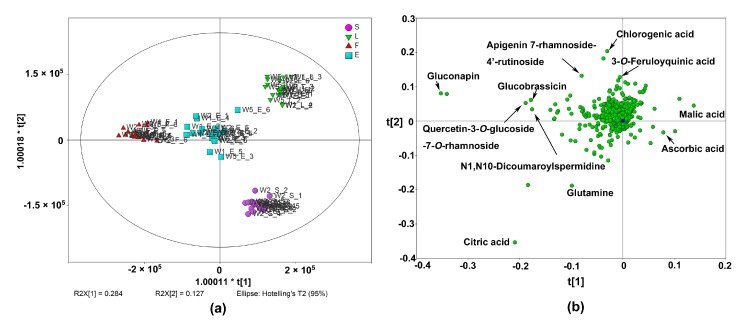
OPLS-DA of different edible parts of Chinese kale including stems (S), leaves (L), inflorescences (F) and all of these parts as a whole (E) (the number after the cultivar name stands for the six independent biological replicates of each edible part of every cultivar). (**a**) Score plot; (**b**) Loading plot.

Four parts had distinctly different metabolite profiles regardless of cultivars (Figure 3a). As expected, the whole edible part (E) group was located in the central area of the score plot. A clear distinction of the leaves and the inflorescences along the first component was observed. Of the features that had high loading scores in component 1 (Figure 3b), two of them were identified as malic acid and ascorbic acid, and these compounds were more abundant in the leaves. Gluconapin, citric acid, glucobrassicin, N1,N10-dicoumaroylspermidine and quercetin 3-*O*-glucoside-7-*O*-rhamnoside were identified on the negative side of the first component. These compounds had higher levels in the inflorescences. The leaves and stems were located at the positive and negative side of the second component, respectively. The positive side of the second component was characterised by chlorogenic acid, 3-*O*-feruloylquinic acid and apigenin 7-rhamnoside-4′-rutinoside. Meanwhile, citric acid and glutamine had higher loading scores on the negative side of the second component, and they were more abundant in the stems than in the leaves.

### 2.4. Nutritive Compounds in Different Edible Parts of Chinese Kale

#### 2.4.1. Primary Metabolites in Different Edible Parts

**Figure 4:**
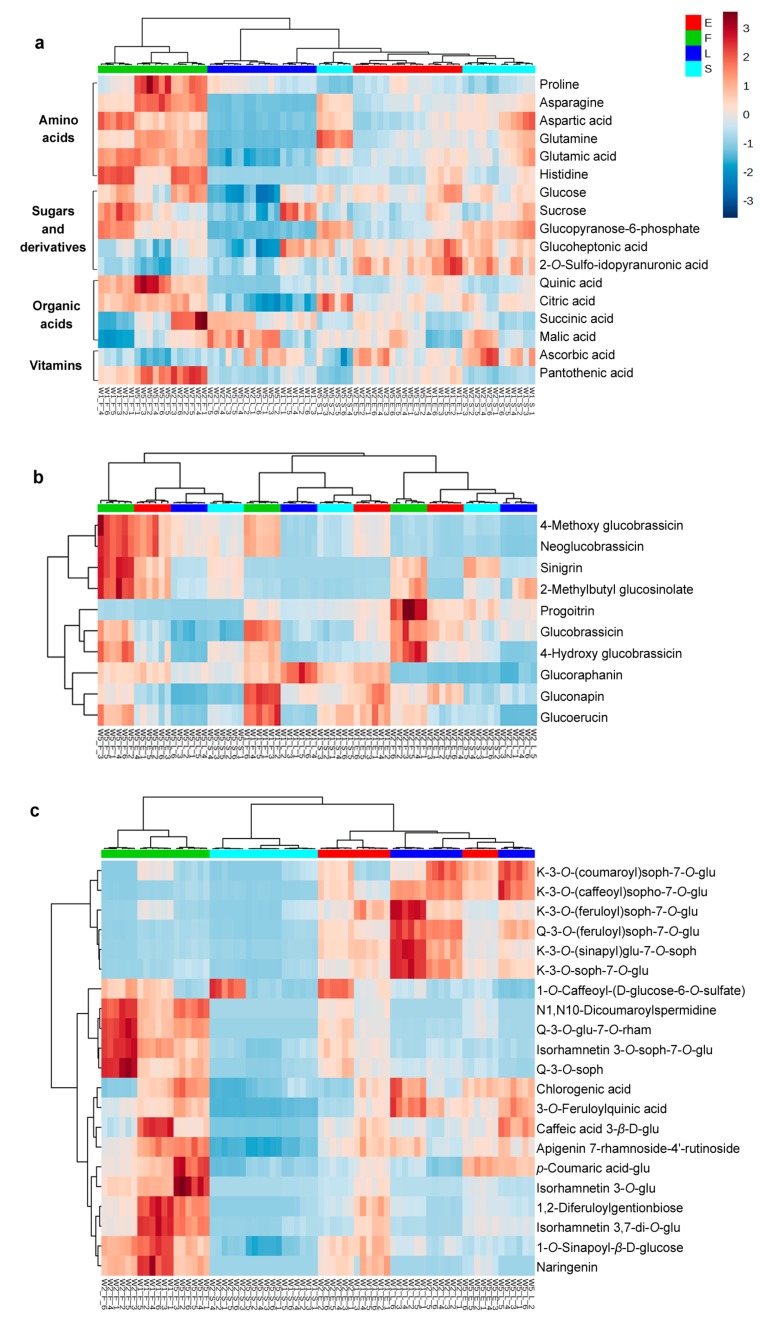
HCA of the primary and secondary nutritive metabolites identified in different edible parts of Chinese kale including stems (S), leaves (L), inflorescences (F) and all of these parts as a whole (E) (each edible part of every cultivar with six independent biological replicates). (**a**) Primary metabolites; (**b**) Glucosinolates; (**c**) Phenolic compounds. glu: glucoside; rham: rhamnoside; K: kaempferol; Q: quercetin; soph: sophoroside.

The content differences of identified nutritive compounds among edible parts were further analysed using HCA (Figure 4). The primary metabolites identified included six amino acids (proline, asparagine, aspartic acid, glutamic acid, glutamine and histidine), five sugars and derivatives (hexose, sucrose, glucopyranose 6-phosphate, glucoheptonic acid and 2-*O*-sulfo-l-idopyranuronic acid) and four organic acids (quinic acid, citric acid, succinic acid and malic acid). Vitamin C and vitamin E (pantothenic acid) were also identified (Figure 4a). As shown in the HCA heatmap, the inflorescences contained the most amounts of amino acids, while the leaves contained the least. Hexose and d-glucose 6-phosphate were higher in the inflorescences and stems. Regarding the identified organic acids, citric acid and quinic acid were much higher in the inflorescences, while malic acid and succinic acid showed cultivar variation in that, generally, they were more abundant in W2 regardless of edible parts. Interestingly, the inflorescences contained the least amounts of vitamin C, whereas this organ had the highest level of vitamin E.

#### 2.4.2. Glucosinolates in Different Edible Parts

**Figure 5:**
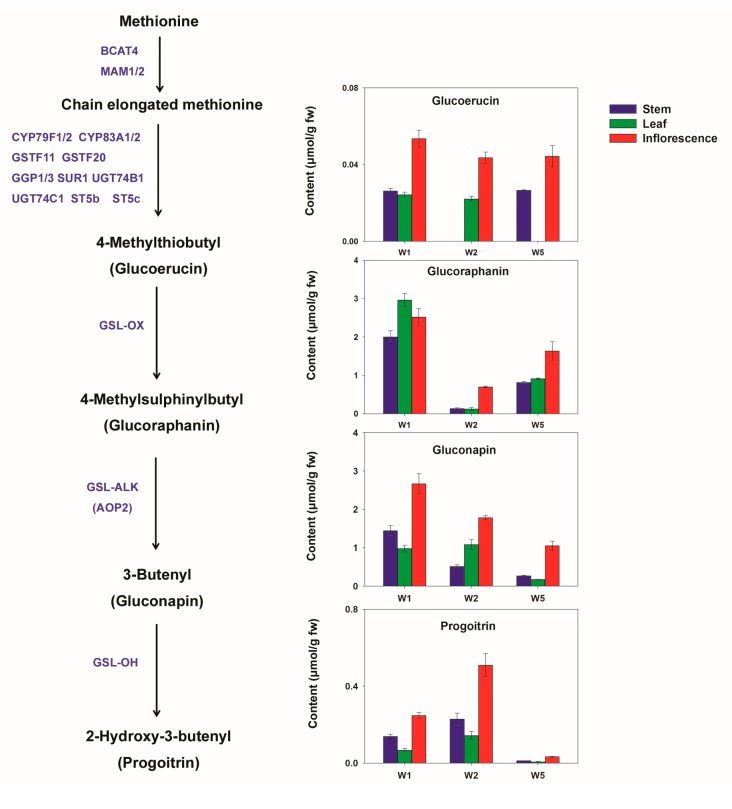
4C aliphatic glucosinolate contents in different edible parts of Chinese kale and their biosynthesis pathway (the related genes are adapted from Wu et al., 2017 [39]). fw: fresh weight.

Obvious differences in GSL contents were observed in different edible parts, although cultivar difference was more significant since different edible parts of the same cultivar were clustered as a group (Figure 4b). Ten GSLs existing in different edible parts of Chinese kale were identified, including six aliphatic GSLs (gluconapin, glucoraphanin, sinigrin, progoitrin, glucoerucin and 2-methylbutylglucosinolate) and four indolyl GSLs (glucobrassicin, 4-hydroxy glucobrassicin, 4-methoxy glucobrassicin and neoglucobrassicin). The contents of these GSLs (except 2-methylbutylglucosinolate) were further quantified. The quantification result of 4C GSLs is shown in Figure 5. Glucoraphanin and gluconapin were the major GSLs in all edible parts. Different from the result of Sun et al. [2], sinigrin and glucoiberin were found in much lower amounts than glucoraphanin in the stems and leaves; meanwhile, glucoraphanin accounted for more than 50% of the total aliphatic GSLs content in the stems and leaves of W1, implying remarkable anticarcinogenic potential of this cultivar. The inflorescences contained higher contents of the identified GSLs than other edible parts (except glucoraphanin in W1). Assuming that GSLs function in defence against herbivores and pathogens, the reproductive organs, including seeds, flowers and fruits, which contribute most to plant fitness, are expected to have the highest concentrations of defence compounds [10].

Recently, de novo transcriptome assembly of Chinese kale and expression profiles of genes involved in GSL metabolism were analysed in 11 tissues [39]. Comparison of the gene expression in the leaves, bolting stems and flower buds showed that most of the genes involved in GSL biosynthesis and breakdown were highly expressed in the senescent leaves and bolting stem skin. This result indicated that GSL biosynthesis and breakdown might happen more intensively in these organs. Meanwhile, our results showed that most GSLs accumulated higher in the inflorescences than in stems and leaves. The amount of glucoraphanin was much higher than its precursor glucoiberin in all edible parts. However, the GSL-OX gene, which is responsible for the oxidation of the methylthiol groups to methylsulphinylalkyl side chains, was not highly expressed in the flower buds, bolting stems and mature leaves [39]. GSL-OH regulates the hydroxylation of alkenylglucosinolates. The GSL-OH expression level was very low in all tissues except root of Chinese kale [39], and it seemed to be partially silenced in Chinese kale [2], as the contents of the hydroxylated product progoitrin were very low in all three edible parts. Further investigations toward the transport of GSLs between organs are needed to clarify the mechanism involved in GSLs accumulation.

#### 2.4.3. Phenolic Acids and Flavonoids in Different Edible Parts

It was shown clearly that the stems contained the least amounts of phenolic compounds except for 1-*O*-Caffeoyl-(*β*-d-glucose 6-*O*-sulfate), which had the highest level in W2 stems (Figure 4c). Most phenolic acids were considerably higher in the inflorescences, but the inflorescences contained much less kaempferol glycoside. Instead, the leaves predominantly contained kaempferol-derived flavonols, which was consistent with the previous reports on *Brassica napus* L. [19]. All the inflorescences were clustered as a group, regardless of cultivars. Similar trends were observed in the stems. The leaves and the whole edible parts showed similar phenolic profiles and were clustered together.

In summary, the cultivar differences in GSLs and phenolic compound profiles were more significant than edible part differences. The inflorescences contained the most abundant GSLs, amino acids, hexose, citric acid, quinic acid, most of the phenolic acids and vitamin E. Chinese kale could be consumed as a leafy vegetable with a rich source of antioxidants [3]. The leaves contained higher levels of kaempferol-derived flavonols, chlorogenic acid, 3-*O*-feruloylquinic acid, apigenin 7-rhamnoside-4′-rutinoside and vitamin C. However, the bolting stems, which are consumed as the most common edible parts, had the lowest levels of most phenolic compounds. From a nutritional perspective, it is recommended that the inflorescences are consumed together with stems and leaves, instead of consuming the stems only.

## 3. Materials and Methods

### 3.1. Plant Materials and Reagents

Seven Chinese kale cultivars, including five white flower cultivars (W1, W2, W3, W4 and W5) and two yellow flower cultivars (Y1 and Y2), were grown in Autumn 2015 in Beijing Vegetable Research Centre (Beijing, China). The stem of W1 is purple, and the stems of other cultivars are green (Figure S1). The grown conditions of Chinese kale followed the descriptions by Sun et al. [2]. The seeds were germinated in a green house. After three weeks, the seedlings with 3 to 5 true leaves were transplanted into the experimental farm. Water, fertiliser, and pesticides were applied as necessary. The plants were harvested when the inflorescences were as tall as the apical leaves. The whole edible parts, including leaves, bolting stems and inflorescences, were harvested (approximately 15 to 20 cm length) for each cultivar. The rosette leaves (L), bolting stems (S), inflorescences (F) and the whole edible parts (E) were then sampled separately from three white flower cultivars (W1, W2 and W5). For each sampling, six independent biological replicates were taken, with each replicate consisting of at least two uniform plants. Fresh plant samples were frozen in liquid nitrogen and stored at −80 °C for further analysis.

High-performance liquid chromatography (HPLC)-grade acetonitrile and methanol were obtained from Honeywell (NJ, USA). HPLC grade formic acid was obtained from J. T. Baker (PA, USA). Ultrapure water was generated using a Milli-Q purification system (Millipore, MA, USA). Sulfatase (EC 3.1.6.1) and DEAE-Sephadex A-25 were obtained from Sigma-Aldrich (St. Louis, MO, USA). Glucotropaeolin was purchased from ChromaDex (Irvine, CA, USA).

### 3.2. Metabolites Extraction

In an attempt to extract as many compounds as possible for the subsequent metabolic analysis, an acidified methanol extraction was used, following the method described by De Vos et al. [15]. Briefly, frozen samples were ground to a fine powder under the protection of liquid nitrogen. Powdered samples (500 mg) were extracted with 1.50 mL of methanol containing 0.1% formic acid (*v*/*v*). The extraction mixture was vortex-mixed and sonicated for 30 min at room temperature and then centrifuged at 5000 rpm/min for 15 min. The supernatant was filtered through a 0.22 μm polyvinylidene fluoride (PVDF) syringe filter before UHPLC-MS/MS analysis.

### 3.3. Chromatographic and Mass Spectrometric Conditions

Chromatographic separation was performed using a DIONEX Ultimate 3000 UHPLC system (Thermo Scientific, Germering, Germany). A Thermo Scientific Hypersil Gold aQ column (100 mm × 2.1 mm, 1.9 μm micron) with a guard column of the same material (10 mm × 2.1 mm) was used. The elution gradient was carried out with a binary solvent system consisting of ultrapure water (eluent A) and acetonitrile (eluent B), both containing 0.05% formic acid (*v*/*v*), at a constant flow rate of 350 μL/min. A gradient profile with the following proportions (*v*/*v*) of solvent B was applied: 0–2 min at 2%, 2–3 min from 2% to 20%, 3–8 min from 20% to 35%, 8–10 min from 35% to 100%, 10–12 min at 100%, and back to 2% in 0.5 min, followed by 4.5 min of re-equilibration. The injection volume was 3 μL, and the column was maintained at 30 °C.

Mass spectrometry analysis was performed on a Q Exactive Quadrupole-Orbitrap mass spectrometer (Thermo Fisher Scientific, Bremen, Germany), which was equipped with a heated electrospray ionization source II (HESI-II) operating in negative ionization mode. The optimised ionization source and MS parameters were set as follows: spray voltage at 3.0 kV, sheath gas at 35 arbitrary units, auxiliary gas at 10 arbitrary units, capillary temperature at 350 °C, heater temperature at 400 °C, and S-lens radio frequency level at 50. The full MS scan range was from *m*/*z* 75 to 1000 with a resolution of 70,000. The MS/MS scan mode resolution was set at 17,500, normalised collision energy (NCE) at 30 v, and stepped NCE at 50%. The automatic gain control (AGC) target values were set at 5 × 10^6^ and 1 × 10^6^ charges for full MS events and MS/MS events, respectively. The maximum injection times for both full MS and data-dependent MS/MS were 100 ms. The mass calibration of the Orbitrap was performed every three days to ensure a mass accuracy of lower than 5 ppm. Instrument control and data processing were carried out by Xcalibur software 2.2 (Thermo Fisher Scientific, Waltham, MA, USA).

### 3.4. Data Processing and Multivariate Analysis

After MS/MS measurement, the raw data were pre-processed using SIEVE 2.2 software (Thermo Scientific, Waltham, MA, USA) for peak extraction, alignment, filtration, normalisation and feature identification. The peaks were picked at a minimal signal to noise ratio of 3. The noise of metabolic data set signals was filtered with 100,000 as the threshold. Only the molecular features present in 100% of the replicates in at least one sample group were considered.

The metabolites were assigned by comparison of the measured accurate mass value with publicly available databases, including mzCloud (https://www.mzcloud.org/), Chemspider (http://www.chemspider.com/), KEGG (http://www.kegg.jp/) and PlantCyc (http://www.plantcyc.org/), as well as by using the retention time, isotopic distribution and fragmentation pattern. High accuracy mass values were used to determine formulas and provide putative identification of metabolites. The mass tolerances lower than 3 ppm were accepted for database searching. For further identification and confirmation of metabolites, the characteristic MS/MS fragments formed with accurate mass measurement were used to elucidate the compound structures. Mass Frontier 7.0 software (HighChem Ltd., Bratislava, Slovakia) was used to obtain the fragmentation pattern of the product ions.

Differences at the feature level between samples were statistically distinguished using an ANOVA test at a significance level of *p* < 0.05 (MetaboAnalyst 3.0, http://www.metaboanalyst.ca) [40]. OPLS-DA was performed to obtain information on differences in the metabolite profiles among cultivars and edible parts using SIMCA Version 13.0.3 software (Umetrics, Umeå, Sweden). A VIP score higher than 1.0 was chosen to select the most discriminant features. Only features with VIP > 1 were assigned. HCA was also performed using MetaboAnalyst 3.0 to compare the metabolite contents in different samples.

### 3.5. Quantitative Analysis of Desulfo-Glucosinolates

Quantification of GSLs in Chinese kale was carried out using the HPLC method described by Chen et al. [41]. Briefly, GSLs were extracted by methanol at 80 °C. The extraction was filtered using a column containing 2 mL DEAE gel. Desulphurisation was carried out by adding 75 μL of sulfatase (EC 3.1.6.1, 29 units) solution and incubated overnight at room temperature. Desulfo-GSLs were eluted with 1.5 mL ultra-pure water and were filtered through a 0.45 μm nylon membrane-filter.

HPLC analyses were performed using a Model LC-20A HPLC instrument (Shimadzu, Kyoto, Japan) equipped with a Model SPD-20AD detector. GSLs separation was performed using a Waters Nova-Pak C18 column (3.9 mm × 150 mm, 5 μm) at room temperature, with a detection wavelength of 229 nm. Mobile phases consisted of 0.05% tetramethylammonium chloride aqueous solution (A) and 0.05% tetramethylammonium chloride with 20% acetonitrile aqueous solution (B) at a flow rate of 1 mL/min. The individual glucosinolate content of each sample was calculated using the internal standard (glucotropaeolin) and expressed as μmol/g of fresh weight.

## 4. Conclusions

The untargeted metabolomics based on the UHPLC-Quadrupole-Orbitrap MS/MS method was applied in the evaluation of Chinese kale nutritional metabolite profiles. The markers featuring different cultivars and edible parts were identified, including glucosinolates, amino acid, organic acids and phenolic compounds. Through comprehensive evaluations of the metabolite profiles, two cultivars, W1 and Y1, were highly recommended from the nutritional point of view. The inflorescences contained considerable amounts of amino acids, glucosinolates and most of the phenolic acids. It is recommended that the inflorescences are consumed together with the stems and leaves. Our systematic study provided more information about the nutrient profiles of Chinese kale, thus revealing that it is a good source of GSLs and phenolic antioxidants. The metabolomics strategy and MS/MS information of metabolites in Chinese kale could be used for further investigations of *Brassicaceae* vegetable nutritional quality and thus provide the foundation for future metabolomics-guided breeding and nutritional quality improvement.

## Figures and Tables

**Table 1:** Identification of the most predominant metabolites in different Chinese kale cultivars and edible parts by ultra-high-performance liquid chromatography (UHPLC)-Quadrupole-Orbitrap MS/MS.

No.	Rt (min)	Compound	Molecular Formula	Experimetal *m*/*z* [M − H]^−^	Theoretical *m*/*z* [M − H]^−^	Accuracy (ppm)	Fragment Ion(s)
Amino acids							
1	0.71	Aspartic acid	C_4_H_7_NO_4_	132.0302	132.0302	0.07	71.0138, 88.0403, 114.0196, 115.0036
2	0.73	Asparagine	C_4_H_8_N_2_O_3_	131.0458	131.0462	2.98	113.0351, 95.0254, 70.0292
3	0.80	Proline	C_5_H_9_NO_2_	114.0555	114.0560	4.60	71.0131, 87.0081
4	0.73	Glutamine	C_5_H_10_N_2_O_3_	145.0614	145.0618	3.03	109.0406, 127.0526, 128.0352, 84.0454
5	0.76	Glutamic acid	C_5_H_9_NO_4_	146.0456	146.0459	1.76	102.0560, 128.0353
6	0.73	Histidine	C_6_H_9_N_3_O_2_	154.0621	154.0622	0.50	93.0457, 110.0723, 137.0356
Sugars and derivatives							
7	0.76	Glucose/Fructose/Fucose/Mannose/Galactose	C_6_H_12_O_6_	179.0558	179.0561	1.58	59.0138, 71.0138, 89.0244, 113.0240
8	0.76	Sucrose	C_12_H_22_O_11_	341.1089	341.1089	0.03	71.0138, 89.0243, 113.0243, 161.0455, 179.0560
9	0.76	Sedoheptulose/d-Mannoheptulose/d-Glucoheptose	C_7_H_14_O_7_	209.0662	209.0667	2.18	85.0288, 87.0081, 129.0188, 159.0296
10	0.68	d-Glucose-6-phosphate/d-Fructose-6-phosphate/d-Mannose-6-phosphate	C_6_H_13_O_9_P	259.0229	259.0224	1.83	78.9590, 96.9696, 138.9801, 199.0013
11	0.73	Glucoheptonic acid	C_7_H_14_O_8_	225.0614	225.0616	0.72	179.0561, 59.0132, 89.0246, 119.0346
12	0.98	2-*O*-Sulfo-l-idopyranuronic acid	C_6_H_10_O_10_S	272.9923	272.9922	0.43	96.9596, 115.0036, 158.0161
Organic acids							
13	0.76	Quinic acid	C_7_H_12_O_6_	191.0555	191.0561	3.05	85.0295, 127.0396, 173.0451
14	0.79	Fumaric acid	C_4_H_4_O_4_	115.0031	115.0037	4.83	71.0138
15	0.80	Malic acid	C_4_H_6_O_5_	133.0141	133.0142	0.97	71.0138, 115.0036
16	0.81	Citric acid	C_6_H_8_O_7_	191.0194	191.0197	1.60	87.0087, 111.0088, 129.0193, 173.0091
17	1.29	Succinic acid	C_4_H_6_O_4_	117.0192	117.0193	0.85	73.0295, 99.0087, 117.0193
Vitamins							
18	0.8	Iso/Ascorbic acid	C_6_H_8_O_6_	175.0246	175.0248	1.14	59.0138, 71.0138, 87.0082, 115.0036
19	4.0	Pantothenic acid	C_9_H_17_NO_5_	218.1028	218.1034	2.61	88.0397, 146.0821
Glucosinolates							
20	0.80	Glucoraphanin	C_12_H_23_NO_10_S_3_	436.0414	436.0411	0.68	96.9596, 178.0169, 259.0129, 274.9895, 372.0429
21	0.81	Sinigrin	C_10_H_17_NO_9_S_2_	358.0277	358.0272	1.43	96.9596, 195.0327, 241.0018, 259.0124, 274.9895
22	0.81	Progoitrin	C_11_H_19_NO_10_S_2_	388.0380	388.0377	0.66	96.9596, 195.0327, 241.0018, 259.0124, 274.9895
23	1.69	Gluconapin	C_11_H_19_NO_9_S_2_	372.0431	372.0428	0.78	96.9596, 195.0327, 241.0018, 259.0124, 274.9895
24	2.21	4-Hydroxyglucobrassicin	C_16_H_20_N_2_O_10_S_2_	463.0497	463.0486	2.29	96.9596, 195.0327, 241.0018, 259.0124
25	4.31	Glucoerucin	C_12_H_23_NO_9_S_3_	420.0468	420.0462	1.43	96.9596, 195.0327, 259.0124, 274.9895
26	4.51	2-Methylbutyl glucosinolate/3-Methylbutyl glucosinolate	C_12_H_23_NO_9_S_2_	388.0747	388.0741	1.49	96.9596, 195.0327, 301.0601, 259.0124, 274.9895, 343.0698
27	4.56	Glucobrassicin	C_16_H_20_N_2_O_9_S_2_	447.0539	447.0537	0.37	96.9596, 259.0124, 274.9895, 241.0018, 205.0433
28	4.57	Glucoaubrietin	C_15_H_21_NO_10_S_2_	438.0544	438.0534	2.28	96.9596, 195.0327, 241.0018, 259.0124, 274.9901
29	4.87	4-Methoxyglucobrassicin	C_17_H_22_N_2_O_10_S_2_	477.0648	477.0643	1.06	96.9596, 195.0327, 259.0124, 314.0433
30	4.76	Gluconasturtiin	C_15_H_21_NO_9_S_2_	422.0592	422.0585	1.74	96.9584, 195.0323, 259.0131, 274.9904
31	4.98	3-Methylpentyl glucosinolate/4-Methylpentyl glucosinolate	C_13_H_25_NO_9_S_2_	402.0898	402.0898	0.06	96.9596, 195.0327, 241.0018, 259.0124, 274.9895, 315.0759
32	5.22	Neoglucobrassicin	C_17_H_22_N_2_O_10_S_2_	477.0644	477.0643	0.22	96.9596, 259.0124, 446.0465
Phenolic acids							
33	4.49	Chlorogenic acid	C_16_H_18_O_9_	353.0881	353.0878	0.87	191.0561, 173.0455, 135.0452
34	4.63	Caffeic acid 3-*β*-d-glucoside	C_15_H_18_O_9_	341.0887	341.0878	2.65	135.0447, 161.0236, 179.0349
35	4.84	3-*O*-Feruloylquinic acid	C_17_H_20_O_9_	367.1036	367.1034	0.49	173.0455, 191.0561, 93.0339
36	6.92	1,2-Diferuloylgentionbiose	C_32_H_38_O_17_	693.2051	693.2036	2.16	175.0389, 193.0497, 217.0500
37	3.23	2-Hydroxy-3- *β*-d-glucopyranosylbenzoic acid	C_13_H_16_O_9_	315.0724	315.0716	2.59	152.0113, 153.0191, 108.0202
38	4.35	4-(*β*-d-Glucopyranosyloxy)benzoic acid	C_13_H_16_O_8_	299.0774	299.0772	0.66	137.0250, 179.0349, 239.0559, 89.0237
39	4.90	1-*O*-Feruloyl-*β*-d-glucose	C_16_H_20_O_9_	355.1039	355.1034	1.35	175.0399, 193.0506
40	4.95	Caffeic acid 3-sulfate/Caffeic acid 4-sulfate	C_9_H_8_O_7_S	258.9921	258.9918	1.15	135.0447, 179.0347
41	4.98	1-*O*-Sinapoyl-*β*-d-glucose	C_17_H_22_O_10_	385.1142	385.1140	0.50	205.0506, 223.0623, 164.0477
42	5.92	N1,N10-Dicoumaroylspermidine	C_25_H_31_N_3_O_4_	436.2247	436.2241	1.27	119.0502, 273.1610, 316.1667
43	4.50	1-*O*-Caffeoyl-(*β*-d-glucose-6-*O*-sulfate)	C_15_H_18_O_12_S	421.0452	421.0446	1.42	96.9584, 161.0233, 179.0347
44	4.86	*p*-Coumaric acid glucoside	C_15_H_18_O_8_	325.0933	325.0929	1.35	117.0331, 145.0282, 163.0400
Flavonoids							
45	6.30	Isorhamnetin 3-*O*-glucoside	C_22_H_22_O_12_	477.1046	477.1038	1.64	314.0432, 315.0510, 285.0405
46	4.87	Isorhamnetin 3,7-di-*O*-glucoside	C_28_H_32_O_17_	639.1590	639.1566	3.67	314.0435, 315.0510, 476.0963, 477.1035
47	4.60	Isorhamnetin 3-*O*-sophoroside-7-*O*-glucoside	C_34_H_42_O_22_	801.2120	801.2095	3.15	314.0434, 315.0506, 476.0964, 477.1034, 639.1575
48	5.12	Quercetin 3-*O*-sophoroside	C_27_H_30_O_17_	625.1420	625.1410	1.57	151.0037, 300.0277, 301.0354, 463.0892
49	5.19	Quercetin-3-*O*- glucoside-7-*O*- rhamnoside	C_27_H_30_O_16_	609.1473	609.1461	2.00	283.0250, 284.0328, 285.0406, 446.0860, 447.0930
50	4.72	Quercetin 3-*O*-(feruloyl)sophoroside-7-*O*-glucoside	C_43_H_48_O_25_	963.2431	963.2412	2.00	191.03395, 284.0326, 285.0400, 609.1469, 801.1893
51	4.59	Kaempferol 3-*O*-sophoroside-7-*O*-glucoside	C_33_H_40_O_21_	771.1998	771.1989	3.74	284.0327, 285.0401, 446.0861, 447.0912, 609.1476
52	4.82	Kaempferol 3-*O*-(sinapyl) sophoroside-7-*O*-glucoside	C_44_H_50_O_25_	977.2593	977.2568	2.54	284.0326, 285.0399, 446.0860, 609.1473, 815.2058
53	4.74	Kaempferol 3-*O*-(caffeoyl)sophoroside-7-*O*-glucoside	C_42_H_46_O_24_	933.2328	933.2306	2.35	284.0327, 285.0403, 609.1472, 771.1789
54	4.94	Kaempferol 3-*O*-(*p*-coumaroyl)sophoroside-7-*O*-glucoside	C_42_H_46_O_23_	917.2375	917.2357	1.98	284.0327, 285.0399, 446.0858, 591.1365, 609.1474, 755.1841
55	4.89	Kaempferol 3-*O*-(feruloyl)sophoroside-7-*O*-glucoside	C_43_H_48_O_24_	947.2485	947.2463	2.37	284.0326, 285.0398, 446.0857, 591.1361, 609.1478, 785.1940
56	6.73	Apigenin 7-rhamnoside-4′-rutinoside	C_33_H_40_O_18_	723.2150	723.2142	1.10	175.0390, 193.0498, 205.0499, 223.0608
57	9.21	Naringenin	C_15_H_12_O_5_	271.0612	271.0612	0.07	151.0037, 177.0193, 119.0502
Other compounds							
58	4.76	3-Acetoxy-4-methoxybenzenesulfonic acid/4-Ethoxy-2-sulfobenzoic acid	C_9_H_10_O_6_S	245.0124	245.0125	0.43	165.0553, 79.9566
59	4.75	Methyl 3,4-*O*-isopropylidene-2-*O*-(methylsulfonyl)pentopyranoside	C_10_H_18_O_7_S	281.0702	281.0700	0.60	96.9594, 201.1127, 281.0702
60	0.80	Glutathione	C_10_H_17_N_3_O_6_S	306.0764	306.0765	0.33	128.0353, 160.0071, 254.0781, 272.0890, 288.0660
61	6.44	*N*-Ethylmaleimide-*S*-glutathione	C_16_H_22_N_4_O_8_S	429.1082	429.1085	0.80	385.1181, 343.1071, 241.0024, 96.9594

## Supplementary Materials

The following are available online: Figure S1: Images and descriptions of the Chinese kale cultivars, Table S1: Glucosinolate contents in Chinese kale whole edible part.

## References

[1] Jahangir M., Kim H.K., Choi Y.H., Verpoorte R. (2009). Health-affecting compounds in *Brassicaceae*. Compr. Rev. Food Sci. Food Saf..

[2] Sun B., Liu N., Zhao Y., Yan H., Wang Q. (2011). Variation of glucosinolates in three edible parts of chinese kale (*Brassica alboglabra* Bailey) varieties. Food Chem..

[3] Sun B., Yan H., Zhang F., Wang Q. (2012). Effects of plant hormones on main health-promoting compounds and antioxidant capacity of Chinese kale. Food Res. Int..

[4] Wolucka B.A., Goossens A., Inzé D. (2005). Methyl jasmonate stimulates the de novo biosynthesis of vitamin C in plant cell suspensions. J. Exp. Bot..

[5] Fahey J.W., Zalcmann A.T., Talalay P. (2001). The chemical diversity and distribution of glucosinolates and isothiocyanates among plants. Phytochemistry.

[6] Traka M., Mithen R. (2009). Glucosinolates, isothiocyanates and human health. Phytochem. Rev..

[7] Keck A.S., Finley J.W. (2004). Cruciferous vegetables: Cancer protective mechanisms of glucosinolate hydrolysis products and selenium. Integr. Cancer Ther..

[8] Cartea M.E., Francisco M., Soengas P., Velasco P. (2010). Phenolic compounds in *Brassica* vegetables. Molecules.

[9] Aires A., Rosa E., Carvalho R. (2006). Effect of nitrogen and sulfur fertilization on glucosinolates in the leaves and roots of broccoli sprouts (*Brassica oleracea* var *Italica*). J. Sci. Food Agric..

[10] Brown P.D., Tokuhisa J.G., Reichelt M., Gershenzon J. (2003). Variation of glucosinolate accumulation among different organs and developmental stages of *Arabidopsis thaliana*. Phytochemistry.

[11] Ciska E., Martyniak-Przybyszewska B., Kozlowska H. (2000). Content of glucosinolates in cruciferous vegetables grown at the same site for two years under different climatic conditions. J. Agric. Food Chem..

[12] Farnham M.W., Wilson P.E., Stephenson K.K., Fahey J.W. (2004). Genetic and environmental effects on glucosinolate content and chemoprotective potency of broccoli. Plant Breed..

[13] Song L., Thornalley P.J. (2007). Effect of storage, processing and cooking on glucosinolate content of *Brassica* vegetables. Food Chem. Toxicol..

[14] Qian H., Sun B., Miao H., Cai C., Xu C., Wang Q. (2015). Variation of glucosinolates and quinone reductase activity among different varieties of Chinese kale and improvement of glucoraphanin by metabolic engineering. Food Chem..

[15] De Vos R.C., Moco S., Lommen A., Keurentjes J.J., Bino R.J., Hall R.D. (2007). Untargeted large-scale plant metabolomics using liquid chromatography coupled to mass spectrometry. Nat. Protoc..

[16] Maldini M., Natella F., Baima S., Morelli G., Scaccini C., Langridge J., Astarita G. (2015). Untargeted metabolomics reveals predominant alterations in lipid metabolism following light exposure in broccoli sprouts. Int. J. Mol. Sci..

[17] Garcia C.J., García-Villalba R., Garrido Y., Gil M.I., Tomás-Barberán F.A. (2016). Untargeted metabolomics approach using UPLC-ESI-QTOF-MS to explore the metabolome of fresh-cut iceberg lettuce. Metabolomics.

[18] Witzel K., Neugart S., Ruppel S., Schreiner M., Wiesner M., Baldermann S. (2015). Recent progress in the use of ߢomics technologies in brassicaceous vegetables. Front. Plant Sci..

[19] Farag M.A., Sharaf Eldin M.G., Kassem H., Abou el Fetouh M. (2013). Metabolome classification of *Brassica napus* L. organs via via UPLC-QTOF-PDA-MS and their anti-oxidant potential. Phytochem. Anal..

[20] Park S., Arasu M.V., Jiang N., Choi S.H., Yong P.L., Park J.T., Al-Dhabi N.A., Kim S.J. (2014). Metabolite profiling of phenolics, anthocyanins and flavonols in cabbage (*Brassica oleracea* var. *Capitata*). Ind. Crops Prod..

[21] Park S.Y., Lim S.H., Ha S.H., Yeo Y., Park W.T., Kwon D.Y., Park S.U., Kim J.K. (2013). Metabolite profiling approach reveals the interface of primary and secondary metabolism in colored cauliflowers (*Brassica oleracea* L. ssp. *Botrytis*). J. Agric. Food Chem..

[22] Mie A., Laursen K.H., Aberg K.M., Forshed J., Lindahl A., Thorup-Kristensen K., Olsson M., Knuthsen P., Larsen E.H., Husted S. (2014). Discrimination of conventional and organic white cabbage from a long-term field trial study using untargeted LC-MS-based metabolomics. Anal. Bioanal. Chem..

[23] Hennig K., de Vos R.C., Maliepaard C., Dekker M., Verkerk R., Bonnema G. (2014). A metabolomics approach to identify factors influencing glucosinolate thermal degradation rates in *Brassica* vegetables. Food Chem..

[24] Millán L., Sampedro M.C., Sánchez A., Delporte C., Antwerpen P.V., Goicolea M.A., Barrio R.J. (2016). Liquid chromatography-quadrupole time of flight tandem mass spectrometry-based targeted metabolomic study for varietal discrimination of grapes according to plant sterols content. J. Chromatogr. A.

[25] Glauser G., Veyrat N., Rochat B., Wolfender J.L., Turlings T.C. (2013). Ultra-high pressure liquid chromatography-mass spectrometry for plant metabolomics: A systematic comparison of high-resolution quadrupole-time-of-flight and single stage Orbitrap mass spectrometers. J. Chromatogr. A.

[26] Kaufmann A. (2014). Combining UHPLC and high-resolution MS: A viable approach for the analysis of complex samples?. TrAC Trends Anal. Chem..

[27] Sun J., Xiao Z., Lin L., Lester G.E., Wang Q., Harnly J.M., Chen P. (2013). Profiling polyphenols in five *Brassica* species microgreens UHPLC-PDA-ESI/HRMS^n^. J. Agric. Food Chem..

[28] Kumaraswamy K.G., Kushalappa A.C., Choo T.M., Dion Y., Rioux S. (2011). Mass spectrometry based metabolomics to identify potential biomarkers for resistance in barley against fusarium head blight (*Fusarium graminearum*). J. Chem. Ecol..

[29] Bijttebier S., Zhani K., D'Hondt E., Noten B., Hermans N., Apers S., Voorspoels F. (2014). Generic characterization of apolar metabolites in red chili peppers (*Capsicum frutescens* L.) by Orbitrap mass spectrometry. J. Agric. Food Chem..

[30] Cao G., Li Q., Cai H., Tu S., Cai B. (2014). Investigation of the chemical changes from crude and processed paeoniae radix alba-atractylodis macrocephalae rhizoma herbal pair extracts by using Q Exactive high-performance benchtop quadrupole-Orbitrap LC-MS/MS. Evid. Based Complement. Altern. Med..

[31] Forcisi S., Moritz F., Kanawati B., Tziotis D., Lehmann R., Schmittkopplin P. (2013). Liquid chromatography-mass spectrometry in metabolomics research: Mass analyzers in ultra high pressure liquid chromatography coupling. J. Chromatogr. A.

[32] Halkier B.A., Du L. (1997). The biosynthesis of glucosinolates. Trends Plant Sci..

[33] Padilla G., Cartea M.E., Velasco P., de Haro A., Ordás A. (2007). Variation of glucosinolates in vegetable crops of *Brassica rapa*. Phytochemistry.

[34] Kim Y.S., Milner J.A. (2005). Targets for indole-3-carbinol in cancer prevention. J. Nutr. Biochem..

[35] Verkerk R., Schreiner M., Krumbein A., Ciska E., Holst B., Rowland I., De Schrijver R., Hansen M., Gerhäuse C., Mithen R. (2009). Glucosinolates in *Brassica* vegetables: The influence of the food supply chain on intake, bioavailability and human health. Mol. Nutr. Food Res..

[36] Halkier B.A., Gershenzon J. (2006). Biology and biochemistry of glucosinolates. Annu. Rev. Plant Biol..

[37] Lin L.Z., Harnly J.M. (2009). Identification of the phenolic components of collard greens, kale, and Chinese broccoli. J. Agric. Food Chem..

[38] Piazzon A., Vrhovsek U., Masuero D., Mattivi F., Mandoj F., Nardini M. (2012). Antioxidant activity of phenolic acids and their metabolites: synthesis and antioxidant properties of the sulfate derivatives of ferulic and caffeic acids and of the acyl glucuronide of ferulic acid. J. Agric. Food Chem..

[39] Wu S., Lei J., Chen G., Chen H., Cao B., Chen C. (2017). De novo transcriptome assembly of Chinese kale and global expression analysis of genes involved in glucosinolate metabolism in multiple tissues. Front. Plant Sci..

[40] Xia J., Sinelnikov I.V., Han B., Wishart D.S. (2015). Metaboanalyst 3.0-making metabolomics more meaningful. Nucleic Acids Res..

[41] Chen J., Li L., Wang S., Tao X., Wang Y., Sun A., He H. (2014). Assessment of glucosinolates in Chinese kale by near-infrared spectroscopy. Int. J. Food Prop..

