# Supplementary material for: Evaluation of the Nutritional Quality of Chinese Kale (Brassica alboglabra Bailey) Using UHPLC-Quadrupole-Orbitrap MS/MS-Based Metabolomics
Source: Molecules. 2017 Jul 27;22(8):1262. doi: 10.3390/molecules22081262 (PMC6152293; doi:10.3390/molecules22081262)
Supplement: Supplementary file 1 [file molecules-22-01262-s001.pdf]

Supplementary Files

Evaluation of the nutritional quality of Chinese kale (*Brassica alboglabra* Bailey) using UHPLC-Quadrupole-Orbitrap MS/MS-based metabolomics

Figure S1. Images and descriptions of the seven Chinese kale cultivars.

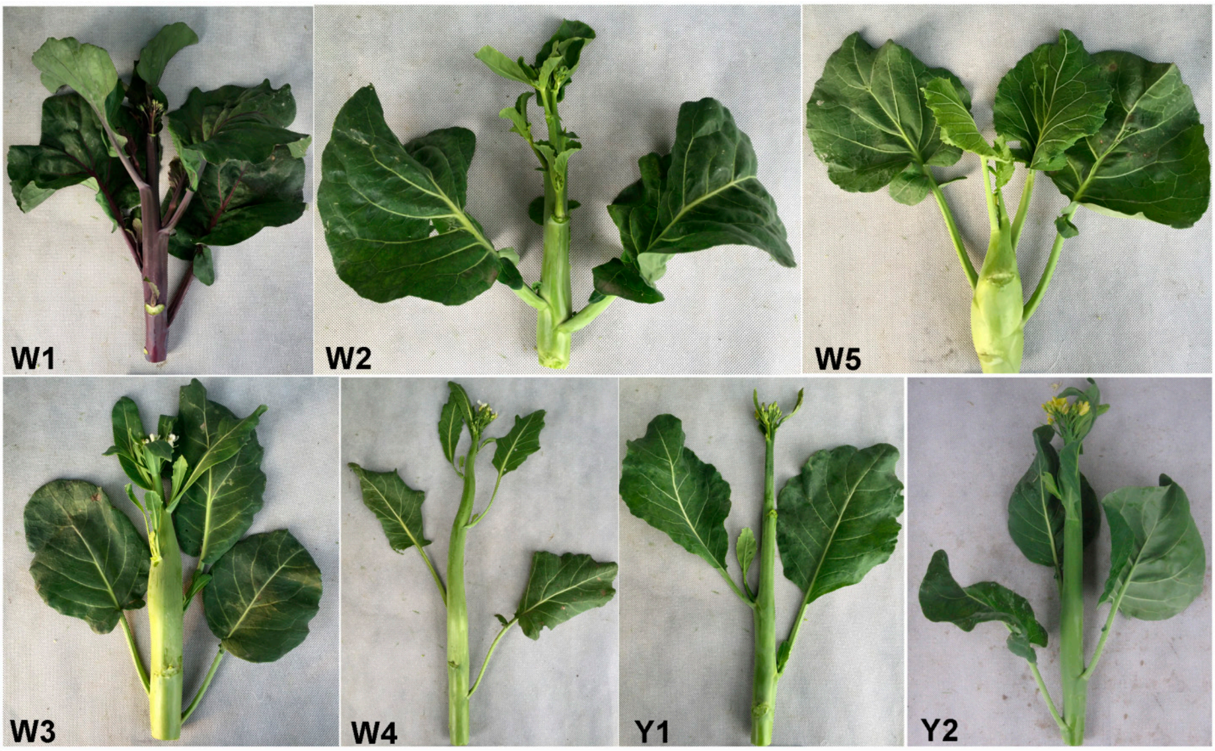

Description	W1	W2	W3	W4	W5	Y1	Y2
Flower color	White	White	White	White	White	Yellow	Yellow
Stem color	Purple	Green	Green	Green	Green	Green	Green
Leaf	Bubble wrinkled surface	Smooth surface	Smooth surface, long petiole	Bubble wrinkled surface, long petiole	Bubble wrinkled surface, long petiole	Smooth surface, long petiole	Bubble wrinkled surface
Origin	Beijing, China	Tokyo, Japan	Shantou, China	Shantou, China	Shantou, China	Guangzhou, China	Shantou, China
Source cultivar of selfing line	Wangtiantaixuan-zhonghua	Jinpincuilv	Jiaxinchneghai	Kunjichenghaicuiyu	Hongjixianggu	Lianjikuaidatian	Shenghetiancui
Note	Inbred line	Doubled-haploid derived from isolated microspore culture	Inbred line	Inbred line	Inbred line	Inbred line	Inbred line

Table S1. Glucosinolate content (µmol/g fresh weight) in Chinese kale whole edible part. Values (mean ± SD, n=3) of the same compound followed by different lowercase letters indicate significant difference (p<0.05).

	W1	W2	W3	W4	W5	Y1	Y2
Gluconapin	2.466±0.088 e	1.667±0.029 c	1.921±0.136 d	0.190±0.028 a	0.239±0.004 a	2.432±0.032 e	1.279±0.022 b
Glucoraphanin	2.910±0.065 d	0.227±0.004 a	0.251±0.012 a	0.574±0.076 b	1.017±0.101 c	3.796±0.140 e	1.024±0.009 c
Glucoerucin	0.041±0.003 c	0.026±0.003 a	nd	0.028±0.003 a	0.025±0.002 a	0.089±0.006 d	0.035±0.002 b
Progoitrin	0.272±0.009 d	0.244±0.006 c	0.437±0.033 f	0.023±0.003 a	0.009±0.002 a	0.379±0.003 e	0.075±0.001 b
Sinigrin	nd	nd	nd	0.266±0.042 a	0.036±0.001 b	nd	nd
Glucobrassicin	0.249±0.019 e	0.240±0.013 e	0.123±0.012 c	0.173±0.022 d	0.054±0.007 a	0.252±0.024 e	0.092±0.005 b
4-Hydroxyglucobrassicin	0.020±0.002 cd	0.015±0.002 bc	0.049±0.005 e	0.024±0.001 d	0.003±0.001 a	0.070±0.006 f	0.013±0.001 b
4-Methoxyglucobrassicin	0.014±0.000 a	0.013±0.000 a	0.022±0.001 b	0.030±0.004 c	0.015±0.002 a	0.034±0.001 d	0.022±0.001 b
Neoglucobrassicin	0.047±0.004 c	0.011±0.002 a	0.016±0.001 a	0.022±0.003 b	0.042±0.003 c	0.070±0.005 d	0.026±0.001 b
Total	6.018±0.184 d	2.444±0.052 b	2.819±0.199 c	1.329±0.176 a	1.439±0.113 a	7.123±0.182 e	2.566±0.038 bc
